# Oscillatory Strain Promotes Vessel Stabilization and Alignment through Fibroblast YAP‐Mediated Mechanosensitivity

**DOI:** 10.1002/advs.201800506

**Published:** 2018-07-15

**Authors:** Shira Landau, Shahar Ben‐Shaul, Shulamit Levenberg

**Affiliations:** ^1^ Department of Biomedical Engineering Technion, Israel Institute of Technology Haifa 3200002 Israel

**Keywords:** endothelial cells, engineered tissues, oscillatory strain, vascularization, YAP

## Abstract

Endothelial cells form the interior layer of blood vessels and, as such, are constantly exposed to shear stress and mechanical strain. While the impact of shear stress on angiogenesis is widely studied, the role of mechanical strain is less understood. To this end, endothelial cells and fibroblasts are cocultured under oscillatory strain to create a vessel network. The two cell types show distinctly different sensitivities to the mechanical stimulation. The fibroblasts, sense the stress directly, and respond by increased alignment, proliferation, differentiation, and migration, facilitated by YAP translocation into the nucleus. In contrast, the endothelial cells form aligned vessels by tracking fibroblast alignment. YAP inhibition in constructs under mechanical strain results in vessel destruction whereas less damage is observed in the YAP‐inhibited static control. Moreover, the mechanical stimulation enhances vessel development and stabilization. Additionally, vessel orientation is preserved upon implantation into a mouse dorsal window chamber and promotes the invading host vessels to orient in the same manner. This study sheds light on the mechanisms by which mechanical strain affects the development of blood vessels within engineered tissues. This can be further utilized to engineer a more organized and stable vasculature suitable for transplantation of engineered grafts.

## 1 Introduction

During vascularization, endothelial cells (ECs) sense two forms of mechanical stimulation, shear stress, as a consequence of blood flow, and mechanical strain, as a consequence of rhythmic heart beating and tissue contraction.^1^, ^2^, ^3^, ^4^ While many studies have focused on the effect of shear stress on vessel mechanotransduction, there is much less information on the effect of mechanical strain on vascularization‐related processes.^4^

Recently, we have shown that vessels orient under mechanical stretch conditions.^5^ While control of vessel orientation is important for vascularization of aligned tissues, such as ligaments, muscle, and the nervous system, the mechanism underlying this orientation under mechanical loading has yet to be established. A better understanding of these processes can enhance the integration progression of vascularized engineered tissues and affect the morphogenesis of anastomosing host vessels upon graft implantation.

YAP, a transcription cofactor involved in Hippo signaling, which regulates organ size by controlling cell proliferation and apoptosis, is also known to be involved in the mechanotransduction of mechanical forces within different cell types.^6^ YAP can be localized in the cytoplasm or translocated to the nucleus, where it associates with TEAD transcription factors.^7^ Previous studies have shown that various forms of mechanical stimulation, such as substrate stiffness, strain, and shear stress, affected YAP localization by actin fiber remodeling.^6^, ^8^, ^9^, ^10^ YAP was shown to be involved in the effect of shear stress on ECs and blood vessels,^10^ however its role in the effect of mechanical strain on vascularization is yet to be established.

In this study, we aimed to investigate the mechanism that regulates organization and remodeling of engineered vessels, and the ECs and mural cells composing them, into a stabilized and organized vessel network in vitro under oscillatory strain. To do so, ECs and fibroblasts were cocultured in gelfoam, a collagen stretchable scaffold, and subjected to oscillatory stretch for 21 d. Our results demonstrated that fibroblasts were more responsive to the stress stimulus as compared to ECs. The mechanical strain induced YAP translocation to fibroblast nuclei, which, in turn, increased their proliferation and differentiation. Moreover, distinct alignment and migration of the fibroblasts cells, alongside vessel development, stabilization, and alignment and a subsequent increase in cell secretion of pro‐angiogenesis cytokines, were observed. Since oscillatory strain was previously shown to affect YAP expression,^9^ we hypothesized that it has a role in transmitting the mechanical stretch signal and affects vessel network development. YAP inhibition, by the addition of verteporfin to the culture media, caused higher damage to the vessels in the mechanically stimulated constructs compared to the vessels in the static control. Subsequently, the implantation of these stable, aligned vascularized structures into a mouse dorsal window chamber resulted in the organization of the penetrating host vessels.

## 2 Results

### 2.1 Vessel Alignment under Oscillatory Strain Is Mediated by Fibroblasts

**Figure 1:**
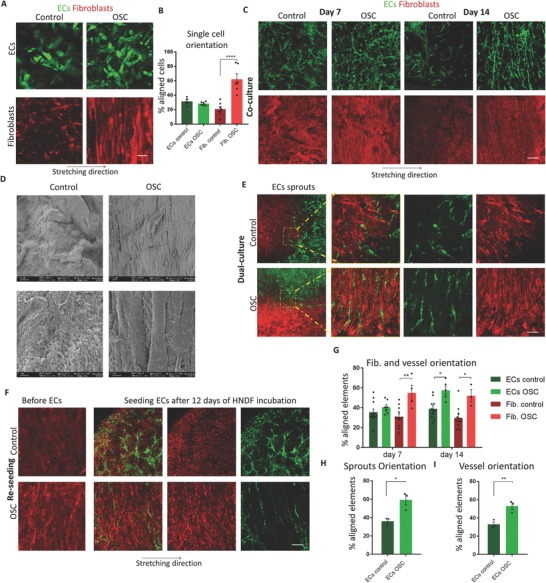
Oscillatory stretch affects cell and network patterning. A) Endothelial cells and fibroblasts were seeded separately on two different scaffolds, and subjected to oscillatory strain. Scale bar = 100 µm. B) Quantification of cell alignment of (A). C) Coculturing ECs and fibroblasts under oscillatory stretch result in aligned vessels, where fibroblasts aligned before (day 7) the forming vessels (day 14). Scale bar = 200 µm. D) SEM images of the oscillatory stretched and control coculture constructs. Scale bar = 2 µm (upper panel) and 10 µm (lower panel). E) ECs and fibroblasts were seeded at either end of a single scaffold and subjected to oscillatory stretch. F) Fibroblasts were subjected to oscillatory stretch for 12 d, after which, ECs were seeded on top of the fibroblasts and seeded under static conditions. ECs formed aligned vessels, following the fibroblasts alignment. Scale bar = 100 µm. G–I) Quantification of cells (C, F), vessel (C, F), and sprout alignment (E). *p < 0.05, **p < 0.01, ***p < 0.001, ****p < 0.0001.

To determine which cell type senses the stretch stimulus, cell alignment of monocultured ECs and fibroblasts on a gelfoam scaffold exposed to oscillatory strain was examined. On day 14, fibroblasts displayed a distinct orientation and alignment, whereas ECs were randomly arranged (**Figure** **1**A,B). However, when fibroblasts were cultured at low densities, no alignment was observed (Figure S1, Supporting Information). When coculturing the two cell types together and applying oscillatory strain, fibroblasts displayed clear alignment on day 7, whereas the endothelial vessel network did not show significant alignment compared to the unstretched control. On day 14 of culturing, the endothelial vessel network displayed the same alignment as the fibroblasts surrounding it (Figure 1C,D,G), hence, ECs only aligned in response to the mechanical strain when fibroblasts were present. To assess whether newly forming endothelial sprouts orient under oscillatory stretch conditions, ECs and fibroblasts were seeded separately at either end of the scaffold. Confocal imaging showed that the endothelial cells formed sprouts only at the point where they met the fibroblasts; the vessel sprouts were aligned and oriented in the same angle as the fibroblasts (Figure 1E,H).

When culturing fibroblast‐seeded gelfoam constructs for 12 d under oscillatory stretch or static conditions and then adding ECs for an additional 5 d without applying stretch, the endothelial vessels followed the fibroblast alignment, and were significantly more oriented when compared to the unstretched control group (Figure 1F,I).

### 2.2 EC and Fibroblast Migration Patterns under Oscillatory Strain

**Figure 2:**
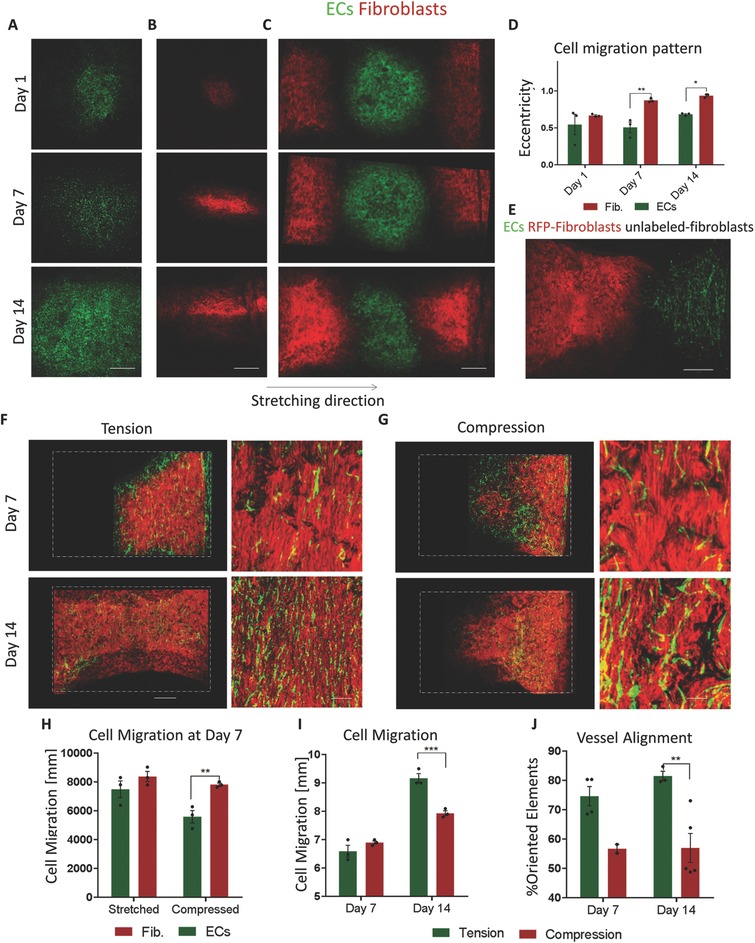
Cell migration throughout the scaffold. A,B) EC (green) and fibroblast (red) migration patterns, as observed on days 3, 7, and 14 of oscillatory strain. Fibroblasts were concentrated in the middle, whereas ECs were spreading throughout the entire scaffold. C) Migration pattern of ECs and fibroblasts when seeded at different scaffold locations. D) Quantification of monocultured EC and fibroblast migration patterns under oscillatory strain. Scale bar = 1000 µm. E) Migration pattern of ECs (green) cocultured with unlabeled fibroblasts (black) and seeded at one scaffold end and of fibroblasts seeded alone (red) at the other end and subjected to oscillatory strain. F,G) Cell migration under oscillatory stretching and compression, white dashed lines represent the scaffolds boundaries. Scale bars = 1000 µm (left panel) and 200 µm (right panel). H) Quantification of EC and fibroblast migration on day 7 under oscillatory stretching and compression. I) Quantification of cell migration on days 7 and 14. J) Vessel, formed under oscillatory stretching and compression, alignment quantification. **p < 0.01, ***p < 0.001.

The differences in cell alignment in response to oscillatory stretch drove us to examine whether cell migration is also affected by oscillatory stretch stimulus. To this end, ECs and fibroblasts were seeded at the center of separate scaffolds and subjected to oscillatory stretch for 14 d. The ECs migrated radially throughout the entire scaffold area, whereas the fibroblasts showed a preference toward migration along the *x*‐axis of the scaffold (**Figure** **2**A,B,D). When culturing the cells together on the same scaffold, ECs in the middle and fibroblasts at both ends, or at the opposite ends, ECs spread radially from the middle and evenly from the side, whereas fibroblast distribution took on a triangle‐shaped pattern (Figure 2C, and Figure S2, Supporting Information). However, when seeding the cells together in the same area of the scaffold, endothelial vessels formed and followed the same migration pattern as the fibroblasts (Figure 2E,F). This phenomenon demonstrated again that under stretch conditions, within a 3D environment, fibroblasts sense and respond to mechanical stimulus, whereas ECs show less responsiveness to this stimulus.

### 2.3 Oscillatory Stretch and Compression Differentially Affect Cell Migration and Alignment

To further understand the influence of stress on cellular behavior, ECs and fibroblast were coseeded on the scaffold ends and subjected to either oscillatory stretch (Figure 2E) or oscillatory compression (Figure 2F) for 14 d. On day 7, ECs and fibroblasts exposed to stretch, migrated together, whereas, under compression, fibroblasts migrated slower than the ECs (Figure 2G). Moreover, at day 14 of culturing under tension condition, both cell types migrated over longer distances when compared to cells in the compressed cultures (Figure 2H); the stretched scaffold was fully covered with cells, whereas the compressed scaffold was half empty. Additionally, vessels were more oriented under stretch conditions (Figure 2I).

### 2.4 Oscillatory Strain Effects on Cell Growth and Network Development Is YAP‐Dependent

**Figure 3:**
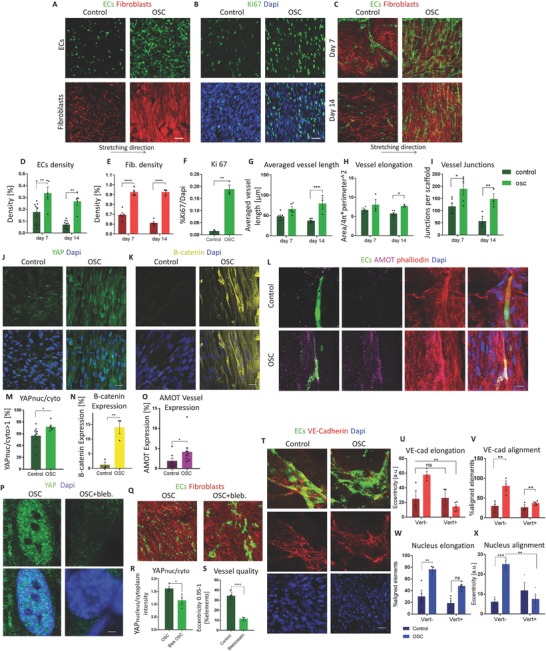
Oscillatory strain effects on cell growth and network development. A) Endothelial cells and fibroblasts were seeded separately on two different scaffolds and cultured for 14 d under oscillatory strain or static control conditions. Cell density was then quantified. Scale bar = 100 µm. B) Ki67 staining of a coculture of ECs and fibroblasts cultured for 14 d under oscillatory strain showed increased cell proliferation when compared to the static control. Scale bar = 50 µm. C) Coculture of ECs and fibroblasts under oscillatory stretch resulted in higher vessel development when compared to the static control. D–I) Quantification of single‐cell density, Ki67 staining, vessel network length vessel elongation, and number of vessel junctions. J–O) Nuclear YAP, cytoplasmic β‐catenin, and AMOT expression in the vessels exposed to oscillatory stretch or static conditions for 14 d. Scale bar = 25 µm. P) YAP staining of fibroblasts within oscillatory stretched and static control vascularized constructs, with the addition of blebbistatin to the culture medium. Scale bar = 2.5 µm. Q) Oscillatory stretched and static control vascularized constructs with the addition of blebbistatin to the culture medium. Scale bar = 100 µm. R) Nuclear YAP and S) vessel quality quantification with and without blebbistatin addition to the culture medium. T) VE‐cadherin staining of vessels within oscillatory stretched and static control vascularized constructs cultured for 7 d with the addition of verteporfin to the culture medium, scale bar = 25 µm. U,V) Quantification of elongation and alignment of VE‐cadherin‐positive elements within verteporfin‐treated constructs. W,X) Quantification of elongation and alignment of DAPI‐positive cells within verteporfin‐treated constructs. *p < 0.05, **p < 0.01, ***p < 0.001, ****p < 0.0001.

In efforts to determine if oscillatory strain affects other characteristics as well, cell proliferation was monitored following exposure of constructs to strain. Under oscillatory strain conditions, both ECs and fibroblast cultures were denser compared to the unstretched control (**Figure** **3**A,D,E). In line with these findings, more Ki67‐positive cells were observed in the oscillatory stretched group (Figure 3B,F). In addition, when coculturing the cells under oscillatory strain conditions, the average vessel length was significantly higher on day 14, vessels were more elongated and the vessel network was more branched, with more vessel junctions (Figure 3C,G–I). When examining the effect of different stretch amplitudes, a positive correlation between stretch amplitude and vessel quality was observed (Figure S3, Supporting Information). To test the involvement of YAP in transmitting the mechanical stretch signal, constructs were stained with YAP antibodies. YAP nucleus/cytoplasm expression ratios in fibroblasts exposed to 14 d of oscillatory stretch conditions were significantly higher when compared to the static control (Figure 3J,M). We previously demonstrated that vessels are localized within the scaffold interior and YAP nuclear expression decreases in the cells located within these areas,^11^ EC YAP expression within the stretched constructs, on day 14, was cytoplasmic and did not show a significant difference from the static control (Figure S4, Supporting Information). In addition, at the same time point, higher cytoplasmic β‐catenin staining was observed in the stretched as compared to the control group (Figure 3K,N). Angiomotin (Amot) is part of the motin family of angiostatin‐binding proteins; it has two isoforms, Amot‐p80 and Amot‐p130. Amot expression is spatially and temporally dependent.^12^ Amot expression within blood vessels was found to be during initial vessel formation, facilitating ECs migration, and is also expressed in mature and stabilized vessels.^13^ Cytoplasmic Amot levels on day 14 were higher within vessels subjected to oscillatory stretching compared to static conditions (Figure 3L,O).

When adding blebbistatin to the culture medium, nuclear YAP levels decreased (Figure 3P,R), blood vessels became disrupted and vessel and fibroblast alignment was lost (Figure 3Q,S). To explore the role of YAP in blood vessel formation, we added verteporfin, which inhibits YAP‐induced transcription,^14^ to the oscillatory stretched constructs and unstretched controls on day 4 of culturing, which led to disruption of vessel network, as indicated by vascular endothelial‐cadherin (VE‐cadherin) disruption, loss of vessel alignment, and loss of nuclear alignment. Less vessel damage was observed in the unstretched control constructs (Figure 3T–X).

### 2.5 Oscillatory Strain Enhances Vessel Development and Stabilization through Secretion of Pro‐Angiogenic Cytokines

**Figure 4:**
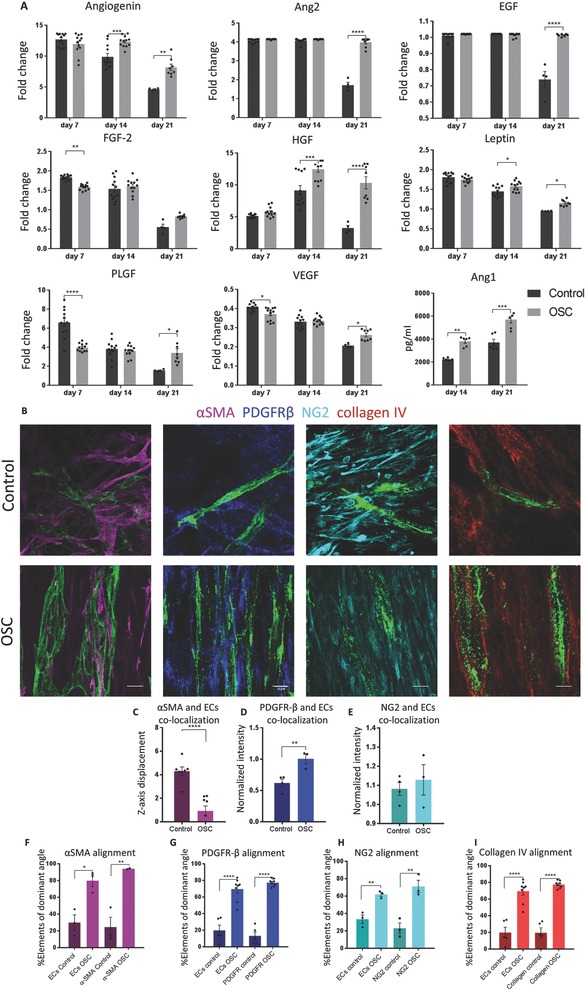
Oscillatory strain affects vessel development and stabilization through secretion of pro‐angiogenic cytokines, fibroblasts differentiation, and collagen VI production. A) Cytokine secretion array was performed on medium collected from oscillatory stretched and static control constructs at days 7, 14, and 21 of culture. Pro‐angiogenic factors were secreted over longer time periods under oscillatory stretch conditions. B) αSMA, PDGFRβ, NG2, and collagen 4 staining of oscillatory stretched and static control constructs cultured for 14 d. C–E) αSMA, PDGFRβ, and NG2 colocalization with ECs vessels quantification. F–I) Markers and blood vessels alignment quantification. Scale bar = 10 µm; *p < 0.05, **p < 0.01, ***p < 0.001, ****p < 0.0001.

Following the observed increase in vessel formation and fibroblast growth under stretch conditions, we set out to examine the growth factors secreted during these processes. Cells in the unstretched static controls showed slightly higher secretion of FGF‐2, VEFG, and PIGF on day 7 compared to the cells within the oscillatory stretched constructs. This trend was reversed on day 14 and further increased by day 21, when angiogenin, angiopoitin2, EGF, HGF, leptin, VEGF, PLGF, and angiopoitin1 secretion were higher in the cells within oscillatory stretched constructs compared to the static control. As sprouting initiation is mainly stimulated by VEGF^15^ and PLGF,^16^ the elevated levels of these cytokines in the static control group at earlier time points led us to hypothesize that the vessel networks form faster under static conditions. However, this vasculature was not stable and degraded faster when compared to the oscillatory stretched constructs (Figures 1C and 3C). In contrast, the higher levels of vessel‐initiating cytokines at later time points and the stabilizing cytokines such as angiopoitin1, which activates mural cells to attach to the forming vessels and which has an important role in maintaining vessel quiescence,^17^ indicate that these vessels reach higher maturation levels and thus are stable over longer time periods (**Figure** **4**).

### 2.6 Mural Cell Differentiation and Collagen IV Secretion under Oscillatory Tensile Forces

Cytokine secretion analysis showed that under oscillatory stretch, vessels develop at a slower rate and are more stable. As mural cell characteristics have been shown to have a major impact on vessel stabilization,^17^ we then examined mural cell differentiation within oscillatory stretched constructs. αSMA and PDGFR‐β are markers for fibroblasts differentiation into myo‐fibroblasts, and are indicators for vessel maturation.^18^ NG2, however, is a marker for fibroblasts, premature cells.^18^ Under stretch conditions, unlike in the unstretched control, both αSMA and PDGFRβ colocalized with the vessel network (Figure 4B–D), whereas NG2 localization was identical in both stretched and control samples (Figure 4E). In addition, the highly aligned morphology of the markers, correlated with vessel alignment (Figure 4F–H). Moreover, under oscillatory stretch conditions, collagen IV alignment was significantly higher and correlated with blood vessel alignment when compared to the static control, likely creating a more stabilized vessel structure (Figure 4B,I).

### 2.7 Implantation of Oscillatory Stretched Constructs Induces Vessel Penetration Alignment In Vivo

**Figure 5:**
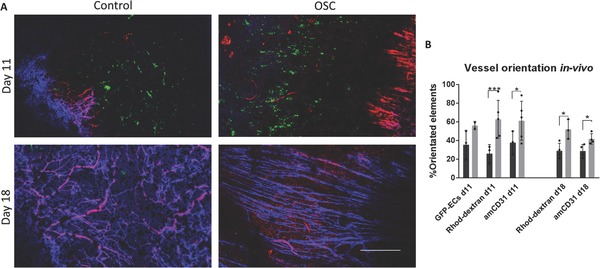
Implantation of oscillatory stretched vascularized grafts into a mouse dorsal window chamber. A) Confocal images of the penetrating host vessels into the implanted oscillatory stretched and static control constructs cultured for 21 d in vitro, stained with rhodamine‐dextran (red) and anti‐mouse CD31 (blue) that were injected via the mouse tail vein. Scale bar = 500 µm. B) Quantification of vessel orientation; *p < 0.05, **p < 0.01, ***p < 0.001.

To determine whether implantation of these mature, organized constructs impact host vessel penetration in vivo, oscillatory stretched and unstretched control constructs were implanted into a mouse dorsal window chamber and then tracked over time. The implantation location was chosen due to its isotropic characteristics, to show whether a highly stable and aligned vasculature can influence the penetration orientation of the host vessels. Vessel penetration into the construct was observed at day 11 after implantation, when the host vasculature anastomosed to and perfused the implanted vessels. By day 18 postimplantation, the graft was fully covered with host blood vessels, which replaced the implanted vessels (**Figure** **5**A). The oscillatory stretched graft showed highly organized vessel penetration on day 11, which further continued by day 18, when the host vasculature was seen aligned in the same orientation as the implanted vasculature, whereas static control graft vessels were organized in a random orientation (Figure 5A,B).

## 3 Discussion

The spontaneous formation of blood vessels in cocultures of endothelial cells and fibroblasts within 3D constructs bears great promise as a solution for more effective tissue integration upon implantation.^19^, ^20^, ^21^ Many recent strategies have concentrated on enhancing vessel stabilization in vitro, such as encapsulating different growth factors within the scaffold, or use of different mural cell types.^22^, ^23^, ^24^ In this study, we present application of oscillatory strain as a simple means of enhancing vessel stabilization and morphogenesis within engineered tissues. The mechanically stimulated vessels were clearly more developed, stabilized, and aligned. Moreover, following their implantation, aligned penetration of the host vasculature was observed.

Application of oscillatory strain on ECs cultured without fibroblasts resulted in randomly oriented and radially spreading cells, in contrast to other works showing that exposure of ECs to oscillatory strain in a 2D culturing context leads to their alignment perpendicular to the stretching direction.^25^, ^26^ In the current work, ECs presented aligned morphology only when cultured with fibroblasts, and may be explained by a differential response to stretch in 3D versus 2D environments. Moreover, when looking at cell migration patterns, ECs spread radially and evenly throughout the entire scaffold, whereas fibroblasts showed a specific migration pattern. Overall, in our system, ECs were less responsive to the stress stimulus as compared to fibroblasts. We hypothesize that the ECM secreted by the cells plays a central role in their responsiveness to strain, since fibroblasts cultured at low densities showed random alignment; only when they secreted sufficient ECM, did they align and the ECs tracked these aligned ECM fibers and align accordingly.

A recent study has shown that 6 h of oscillatory stretch of human mammary epithelial cells resulted in increased nuclear YAP levels, which was shown to correlate with cell proliferation.^9^ Another work has shown that culturing cells on stiff substrates resulted in a flat‐spread morphology with higher proliferation rates and an increase in nuclear YAP, as opposed to soft substrates, which resulted in a rounded cell shape, decreased proliferation rates, and more cytoplasmic YAP.^27^ These results are in correlation with our findings, which demonstrated a significant increase in cell proliferation rates and in nuclear YAP under oscillatory stretch. Moreover, we showed that addition of verteporfin, an inhibitor that abolishes YAP and TEAD interaction, thereby inhibiting YAP‐induced transcription,^7^ was more destructive in the oscillatory‐stretched group, as shown by damaged VE‐cadherin within these constructs and loss of vessel alignment.

Vessel stabilization is achieved by recruitment of mural cells and the formation of the basement membrane. During this stage, EC proliferation decreases, and mural cells differentiation increases.^18^, ^28^, ^29^ These processes are facilitated by secretion of various cytokines. Angiopoietin (Ang) 1 stabilizes the vessel by tightening the contacts between the recruited mural cells and the ECs, thereby decreasing vessel leakiness.^17^, ^30^ Our results show higher secretion of Ang1 in the oscillatory stretched group, suggesting that this mechanical stimulation increases vessel stability. This conclusion is also supported by the higher number of αSMA‐ and PDGFRβ‐positive cells seen surrounding the oscillatory stretched vessels. In the coronary artery system, αSMA expression was found near large and mature arteries,^18^ and is considered a marker of mural cells differentiation into smooth‐muscle cells and hence, vessel maturation.^29^ In addition, collagen IV deposition was aligned in the same manner as the cells, leading us to conclude that endothelial microvessels forming under oscillatory‐stretch are more mature and stable, and surrounded by aligned ECM.

In order to engineer a complex tissue, there is a need to pattern the vascular system within it. This has been previously achieved using micropatterning techniques, which was applied to fabricate endothelial cords, which have facilitated host penetration alignment.^31^, ^32^ In our in vivo model designed to assess whether the implanted construct can influence host vessel penetration orientation, the oscillatory‐stretched constructs were implanted into the dorsal area, which contains randomly aligned vasculature. Oscillatory‐stretched constructs were grown for 21 d in vitro, a time point which showed the most developed vasculature upon implantation. We hypothesized that upon implantation, the construct components, aligned vessels, surrounding fibroblasts, and ECM serve as tracks for the invading vessels, which by day 18 postimplantation covered the entire graft and took on the same orientation as the implanted vessels.

## 4 Conclusion

**Figure 6:**
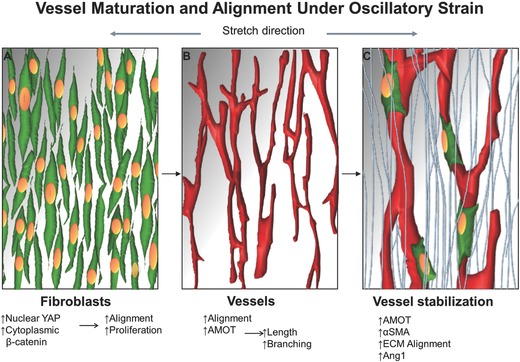
Model of vessel stabilization and alignment under oscillatory stretch. A) Oscillatory strain is first sensed by the fibroblasts cells (green cells), which respond by translocating YAP to the nucleus, and by an increase in cytoplasmic β‐catenin which results in alignment and increased proliferation. B) Next, endothelial cells form aligned vessels according to the fibroblast alignment; this vasculature is more complex and longer when compared to the static control and associated with AMOT upregulation. C) Then, vessel stabilization occurs: fibroblasts differentiate and express more αSMA and are recruited to the vessels (green cells); the ECM aligns and stabilizes the vessels (light blue fibers); Ang1, a vessel stabilization growth factor, is secreted.

These results led us to propose a new mechanism for vessel network development under oscillatory strain (**Figure** **6**): application of oscillatory stretch enhances mural cell alignment and proliferation, translocation of YAP into the nucleus, and higher expression levels of β‐catenin. In turn, more ECM is produced, and is oriented in the same direction as the fibroblasts. ECs, which are less responsive to stretch stimulus, form aligned vessels by following the fibroblast and ECM alignment. Mechanical uncoupling of the cells, by adding blebbistatin to the culture medium, results in loss of fibroblast alignment and hence, loss of vessel alignment. Moreover, when inhibiting cell proliferation and ability to respond to the mechanical stimulus, by inhibiting YAP, both fibroblasts and vessels are affected. The stretch stimulus also increases mural cell differentiation, thereby increasing vessel stability and alignment, as indicated by the secretion of stabilizing growth factors. Upon implantation of the vascularized construct, invading vessels align in the same direction as the implanted vessels. These findings provide a better understanding of the role of mechanical strain on cell development, differentiation, and morphogenesis during the vascularization process. This will enhance engineered vascularized tissue stability and utility, and significantly contribute to the field of tissue engineering and regenerative medicine.

## 5 Experimental Section

*Scaffolds and Mechanical Stimulation*: Gelfoam scaffolds (Gelfoam compressed, Pfizer) were cut to 1 cm × 0.5 cm. Mechanical stimulation was applied on the constructs 1 d postseeding, using an EBERs TC‐3 bioreactor, with uniaxial oscillatory stretching or compression (sinusoidal) of 20% strain and 1 Hz frequency, was applied for 21 d.

*Cell Culture and Inhibitors*: Human adipose microvascular endothelial cells (HAMECs; ScienceCell) lentivirally transduced with ZsGreen fluorescent protein, were grown in endothelial cell medium (ScienceCell) supplemented with 5% fetal bovine serum (FBS) (ScienceCell) and endothelial cell growth supplement (ScienceCell), and were used for 5–9 passages. Neonatal normal human dermal fibroblasts expressing red fluorescent protein (HNDFs‐RFP) (Angio‐Proteomie) were grown in Dulbecco's modified Eagle medium (DMEM) (Gibco), supplemented with 10% FBS (HyClone), 1% nonessential amino acids (NEAAs), 0.2% β‐mercaptoethanol (Sigma‐Aldrich), and 1% penicillin‐streptomycin solution (PEN STREP) (Biological Industries). 3D vascularized constructs were obtained by coseeding endothelial cells (HAMECs, 3 × 10^5^ cells) and support cells (HNDFs, 0.6 × 10^5^ cells) on the gelfoam scaffolds, by mixing the cells, seeding them in a small volume of medium (20 µL), and incubating them for 15 min before adding medium. Verteporfin (1 × 10^−6^ m, Biotest) was added to the culture medium 3 d postseeding, and then constructs were cultured for 7 d. Alternatively, blebbistatin (50 × 10^−6^ m, Sigma‐Aldrich) was added to the culture medium 3 d postseeding, constructs were cultured for 14 d.

*Whole‐Mount and Cryosection Immunofluorescence Staining*: Constructs were fixated in paraformaldehyde (4%) for 20 min, and then permeabilized with 0.3% Triton X‐100 (Bio Lab Ltd.) for 10 min. Constructs were then washed with PBS and immersed in BSA solution (5%; Millipore) overnight. Samples were then incubated with the following primary antibodies overnight at 4 °C: mouse anti‐human Ki‐67 (1:20; DAKO), mouse anti‐human αSMA (1:50; DAKO), goat anti‐human PDGFRβ (1:50; R&D), mouse anti‐human NG2 (1:100; Santa‐Cruz), mouse anti‐human collagen IV (1:500, Sigma‐Aldrich), goat anti‐human VE‐cadherin (1:100; Santa Cruz), mouse anti‐human YAP (1:100; Santa Cruz), rabbit anti‐human β‐catenin (1:100; Sigma‐Aldrich), or mouse anti‐human AMOT (1:100; Santa Cruz). Constructs were then treated with Cy3‐labeled (1:100; Jackson Immunoresearch Laboratory), Cy5‐labeled (1:100; Jackson Immunoresearch Laboratory), or Alexa‐488‐labeled (1:400; ThermoFisher Scientific) secondary antibodies and DAPI (Sigma‐Aldrich), for 2 h, at room temperature.

*Scanning Electron Microscopy (SEM) Imaging*: Scaffold morphology was examined using a SEM (Zeiss Ultra‐Plus FEG‐SEM). Scaffolds without cells were carbon‐coated using a Polaron carbon coater (Quorum Technologies). Cell‐embedded scaffolds were fixed in 2.5% (vol/vol) glutaraldehyde in 0.1 m cacodylate buffer (Sigma‐Aldrich) for 5 min, followed by dehydration in a gradient of 70, 85, 95, and 100% ethanol, with a 5 min incubation in each solution. Scaffolds were then immersed in hexamethyldisilazane (Sigma‐Aldrich) for 5 min and air‐dried at room temperature, before being coated with a gold‐palladium mixture using a Polaron gold coater.

*Cytokine Array and ELISA*: Medium was collected on days 7, 14, and 21 postseeding. Cytokine quantification was measured using the Human Angiogenesis Array GS1 (RayBiotech). Ang1 was quantified using the Human ANGPT1 ELISA kit (RayBiotech).

*Construct Imaging and Image Analysis*: Whole vascularized constructs were imaged with a confocal microscope (LSM700, Zeiss), using 2.5×, 5×, 20×, and 63× oil immersion lenses. Imaris software (BITPLANE) was used to detect YAP, PDGFRβ, and NG2 localization in the 3D image. All other image analyses were quantified using self‐written algorithms in MATLAB: all images were transformed into a binary image and then analyzed using different algorithms.

Cells, nuclei, vessels, and collagen IV orientation were determined using the orientation module in the regionprops function in MATLAB software. Cell migration pattern, VE‐cadherin morphogenesis, and vessel quality under blebbistatin treatment were measured by the eccentricity parameter using regionprops algorithm in the MATLAB software. Ki67 pixel density was calculated by dividing Ki67 staining‐positive pixels by DAPI staining‐positive pixels. AMOT vessel expression was calculated by determining the number of pixels with both a positive vessel (ZsGreen) signal and AMOT‐positive signal.

*Animal Experiments*: Animal studies and surgical procedures were conducted according to protocols approved by the Technion animal ethics committee. Dorsal window chamber assembly followed the protocol by Palmer et al.^33^ Window parts were soaked in 70% ethyl alcohol for 1 h and washed three times with PBS. Athymic nude mice (≈30 g, 7–9 weeks; Harlan Laboratories) were anesthetized via intraperitoneal injection with a mixture of ketamine (100 mg kg^−1^)‐xylazine (10 mg kg^−1^), delivered via a 30gauge needle. On the following 2 d, mice were subcutaneously injected with buprenorphine (0.05 mg kg^−1^) every 12 h. To create an aseptic working area, the dorsal region of the mouse torso and the entire tail were cleaned with chlorhexidine solution and iodine. To assemble the window, mouse skin was stretched and sutured to a stabilizing device using 4‐0 silk sutures, after which, two window frames were sutured to each other using 4‐0 silk sutures. The skin was then cut and removed along the circumference of the window and then the area was washed with warm saline and covered with a 13 mm cover slip glass (Electron Microscopy Sciences, PA, USA), and fixed with a snap ring. The mice were monitored daily to assess general health.

*Scaffold Implantation*: 2 or 3 d after implantation of the dorsal skinfold window chamber, mice were anesthetized via intraperitoneal injection of a mixture of ketamine (100 mg kg^−1^)‐xylazine (10 mg kg^−1^), delivered via a 30 gauge needle. The snap ring and the cover slip glass were removed, followed by a saline wash of the tissue. The scaffold was then placed in the middle of the window groove and then covered with a new cover slip. The window was then closed with the snap ring. Mice were monitored daily and imaged using intravital microscopy.

*Intravital Imaging*: Intravital microscopy (LSM700 confocal microscope, Carl Zeiss) was performed on days 11 and 18 postimplantation. To track the host vasculature in the mice within the graft area, Alexa flour647 anti‐mouse CD31 (Biolegend) was intravenously injected via the tail vein. The antibody was allowed to circulate for 30 min before mice were anesthetized via intraperitoneal injection of ketamine (100 mg kg^−1^)‐xylazine (10 mg kg^−1^), delivered using a 30 gauge needle. Tetramethylrhodamineisothiocyanate‐dextran, (average MW 155 000, Sigma‐Aldrich) was then intravenously injected through the tail vein. Mouse body temperature was maintained during the entire imaging session with a heating chamber.

*Statistical Analysis*: Presented data include the mean ± standard deviation. Two‐way analysis of variance (ANOVA) was performed to examine the influence of two independent categorical variables, followed by Bonferroni's multiple comparison tests. Results were considered significant for *p* < 0.05. Statistical analysis was performed using GraphPad Software, a computerized statistical program. All analyses were performed in at least biological triplicates.

## Conflict of Interest

The authors declare no conflict of interest.

## Supporting information

Supplementary
