# Supplementary material for: Oscillatory Strain Promotes Vessel Stabilization and Alignment through Fibroblast YAP‐Mediated Mechanosensitivity
Source: Adv Sci (Weinh). 2018 Jul 15;5(9):1800506. doi: 10.1002/advs.201800506 (PMC6145399; doi:10.1002/advs.201800506)
Supplement: Supplementary file 1 — Supplementary [file ADVS-5-1800506-s001.pdf]

Copyright WILEY-VCH Verlag GmbH & Co. KGaA, 69469 Weinheim, Germany, 2018.

ADVANCED SCIENCE
Open Access

Supporting Information

for *Adv. Sci.*, DOI: 10.1002/advs.201800506

Oscillatory Strain Promotes Vessel Stabilization and Alignment through Fibroblast YAP-Mediated Mechanosensitivity

*Shira Landau, Shahar Ben-Shaul, and Shulamit Levenberg**

Copyright WILEY-VCH Verlag GmbH & Co. KGaA, 69469 Weinheim, Germany, 2016.

Supporting Information

Oscillatory Strain Promotes Vessel Stabilization and Alignment Through Fibroblast YAP-Mediated Mechanosensitivity

*Shira Landau, Shahar Ben-Shaul and Shulamit Levenberg**

Figure S1

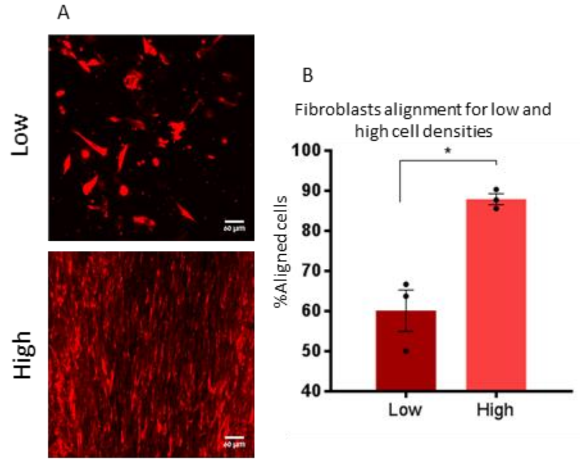

Figure S1: Fibroblast alignment depends on cell density (A) Confocal images of fibroblasts initially seeded at a density of 2000 cells per scaffold (low) and 60,000 cells per scaffold (high) and then cultured for 14 days. **(B)** Quantification of cell alignment, *p<0.05.

Figure S2

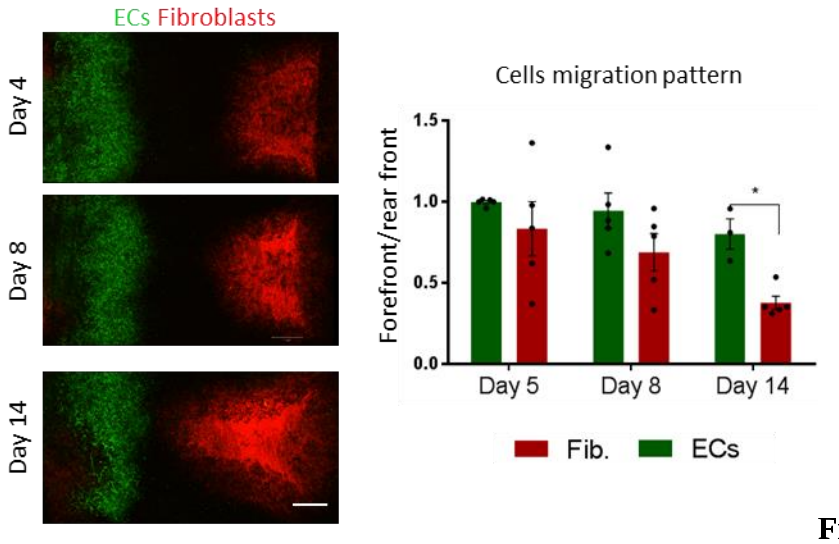

Figure S2: Cell migration patterns. Confocal images of ECs and fibroblasts seeded at opposite scaffolds ends on days

4, 8 and 14, and quantification of the ratio of the fore and rear front length. Scale bar=1000µm.

Figure S3:

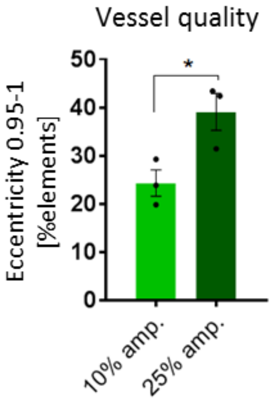

Figure S3: Higher strain amplitudes increases vessel quality. Vessel quality measured by the eccentricity parameter, on day 14, for 10% and 25% amplitudes. *$p<0.05$.

Figure S4:

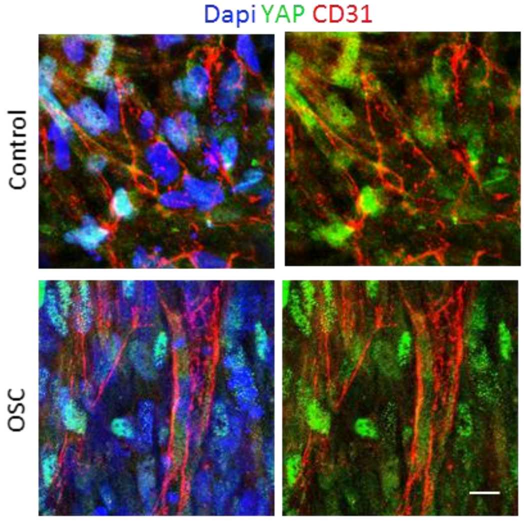

Figure S4: YAP nuclear expression within EC vessels. Confocal images of co-culture constructs conttaining ECs and fibroblasts cultured for 14 days and stained for YAP and CD31 and with DAPI; Scale bar=50µm.
